# Torque Control During Intrusion on Upper Central Incisor in Labial and Lingual bracket System - A 3D Finite Element Study

**DOI:** 10.4317/jced.54461

**Published:** 2018-01-01

**Authors:** Tejas R. Pol, Meghna Vandekar, Anuradha Patil, Sanjana Desai, Vikram Shetty, Saptarshi Hazarika

**Affiliations:** 1 Assistant professor Department of orthodontics, YMT Dental College and Hospital, kharghar, India; 2 HOD < Professor ,Department of orthodontics, YMT Dental College and Hospital, kharghar; 3 Associate Professor Department of conservative and Endodontics, Mgm dental college and hospital. New mumbai, India; 4 Associate Professor, Karnavati dental college, Ahemdabad ,Gujrat, India; 5 Professor Department of orthodontics, YMT Dental College and Hospital, kharghar, India; 6 Lecturer Department of orthodontics, YMT Dental College and Hospital, kharghar, India

## Abstract

**Background:**

The aim of present study was to investigate the difference of torque control during intrusive force on upper central incisors with normal, under and high torque in lingual and labial orthodontic systems through 3D finite element analysis.

**Material and Methods:**

Six 3D models of an upper right central incisor with different torque were designed in Solid Works 2006. Software ANSYS Version 16.0 was used to evaluate intrusive force on upper central incisor model . An intrusive force of 0.15 N was applied to the bracket slot in different torque models and the displacements along a path of nodes in the upper central incisor was assessed.

**Results:**

On application of Intrusive force on under torqued upper central incisor in Labial system produce labial crown movement but in Lingual system caused lingual movement in the apical and incisal parts. The same intrusive force in normal-torqued central incisor led to a palatal movement in apical and labial displacement of incisal edge in Lingual system and a palatal displacement in apical area and a labial movement in the incisal edge in Labial systemin. In overtorqued upper central incisor, the labial crown displacement in Labial system is more than Lingual system.

**Conclusions:**

In labial and lingual system on application of the same forces in upper central incisor with different inclinations showed different responses. The magnitudes of torque Loss during intrusive loads in incisors with normal, under and over-torque were higher in Labial system than Lingual orthodontic appliances.

** Key words:**FEM, lingual orthodontics, intrusion, torque control, labial bracket systems

## Introduction

Lingual Orthodontics (LiO), one of the most popular and rapidly growing esthetic orthodontic technique in the world and today is as popular as the labial technique among orthodontists and patients all over the world.

However, there are many clinical and biomechanical differences between the two techniques (1-4). It is essential for better orthodontic results to completely understand the biomechanical differences of torque control of the maxillary incisors during retraction between LiO and LaO.

Hence, simply following the labial mechanics blindly and applying it in LiO can be inappropriate and may result in a less than optimal treatment results.

Nevertheless, lingual appliances have their own peculiar biomechanics, distinct from that of conventional orthodontics, and special care must be taken in their application.

From biomechanical point of view, one difference of labial and lingual systems would be the ability in torque control. According to Demling *et al.* in a comparative analysis of slot dimension claimed that slot precision in LiO is an important factor for minimal play of wire which results in three-dimensional control (5). In contrast, Liang *et al.* demonstrated that loss of torque control during retraction of upper incisors is more probably happen in extraction cases in LiO treatment. During retracting incisors in LiO, the control of incisor torque is so important that since when lingual crown tipping appears, it is much more difficult to correct than in Labial orthodontics( LaO) (6).

Clinically it is very difficult to evaluate the stress induced at various locations within the root by different types of orthodontic tooth movement. Although a variety of traditional analytical and experimental methods for analyzing dental stresses, such as photoelasticity, interferometric holography, and strain gauges, have given information on the mechanism of orthodontic tooth movement, they have been unable to clarify the microenvironmental changes around the periodontal ligament (PDL) and within the bone (7).

However, the finite element method (FEM) described by Zienkiewicz has been used to investigate a wide range of dentistry topics including tooth structure, (8,9) biomaterials and restorations (8-10) and dental implants and root canals (8,9) and may elucidate the reaction of the teeth, periodontal ligament, alveolar bone, etc. to orthodontic loading. FEM is a mathematical method in which the shape of complex geometric objects and their physical properties are computer-constructed. In order to capitalize on this powerful computational tool, we set out to make 3D FEM models. The purpose of this study was to evaluate and assess the torque control in labial and lingual orthodontic systems using light orthodontic forces during intrusion of normal, under and high torque upper central incisors in lingual and labial orthodontic systems using finite element method.

## Material and Methods

Six 3D models of an upper right central incisor and its supporting structure were designed in Solid Works 2006 The tooth was modeled according to Ash’s dental anatomy (Nelson. 2009) When designing the 3D FEM model, smaller elements were assigned to the areas with the potential for high-stress gradients, such as teeth, PDL, and adjacent alveolar bone.

**Figure 1:**
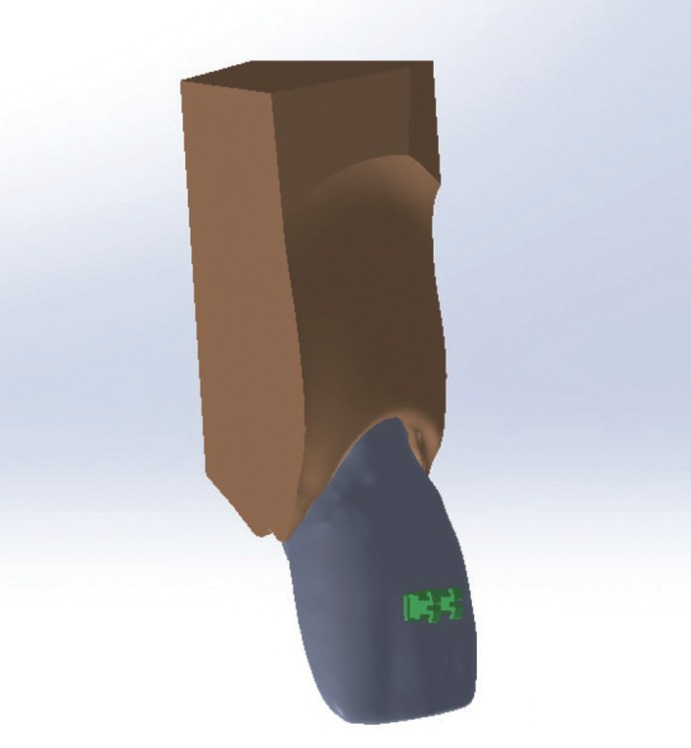
3D model of teeth with labial bracket.

**Figure 2:**
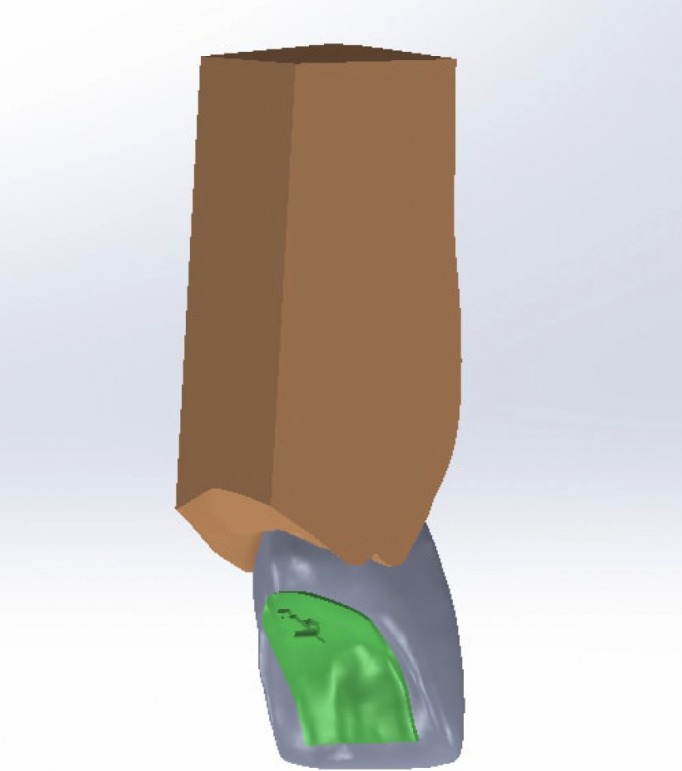
3D model of teeth with lingual bracket

The models were same except 2 points Torque:-under-torqued, normal-torqued, high torqued upper central Incisors, (Figs. 1,2).

**Figure 3:**
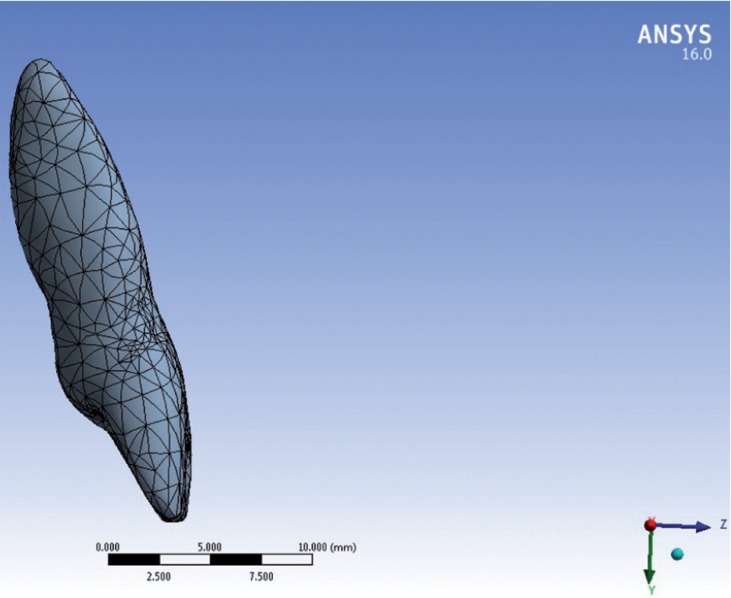
3D meshed model.

The models were transferred to the ANSYS Version 16.0. Boundary conditions restricted displacements of the base of the models in all direction to prevent their rigid body motion. Mechanical properties ( Table 1) were then applied and the models were meshed with 5119 nodes and 9367 elements (Fig. 3). (There was a slight difference between node and element numbers between models) A force of 0.15 N which is the appropriate magnitude of intrusion force 11 was applied to the bracket slot in different models and the displacements along a path of nodes in the upper central incisor was assessed.

**Table 1:**
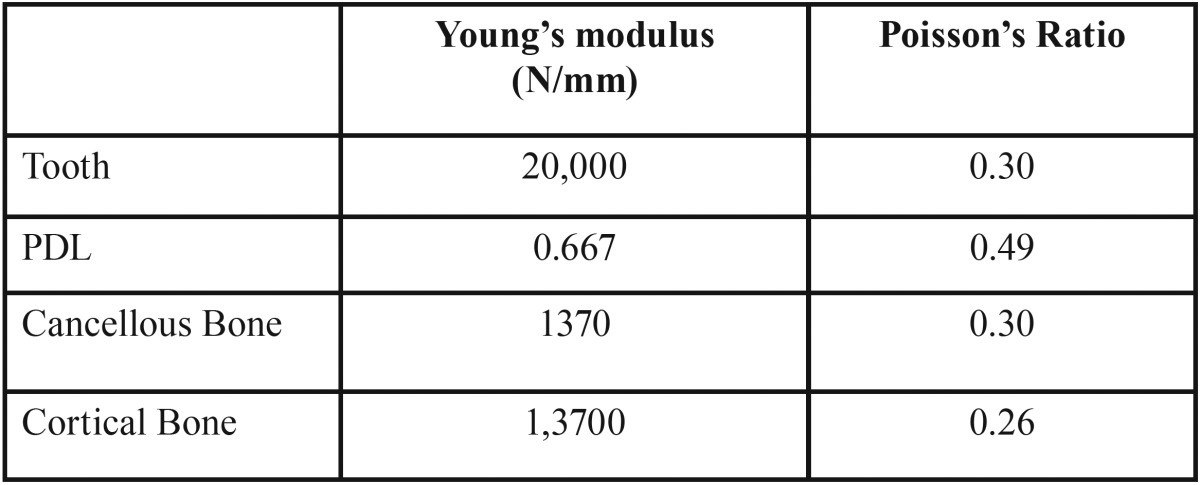
Mechanical properties.

## Results

Negative findings represent a palatal displacement and positive ones show a labial movement.

**Figure 4:**
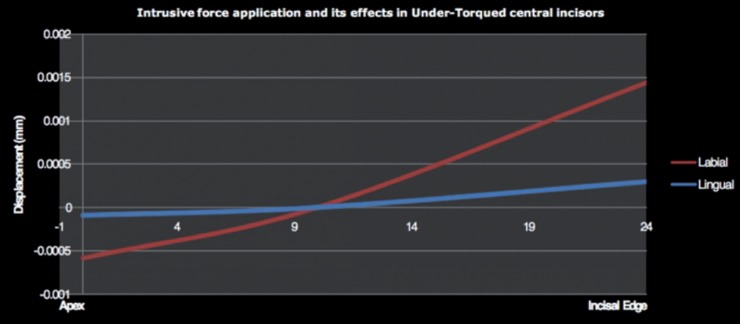
Tooth displacement in under-torqued upper central incisor

Intrusive force of 0.15 N in under-torqued model in Lao induced labial crown movement 0.00144mm) and palatal root displacement (-0.000583mm) but in Lio caused lingual movement in the apical (-0.00009mm) and incisal parts (0.000301mm) of the tooth (Fig. 4).

**Figure 5:**
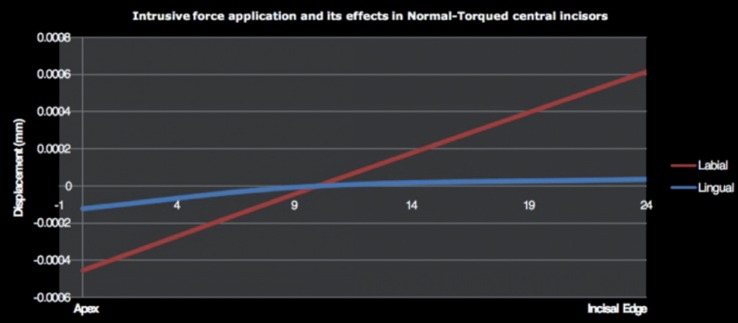
Tooth displacement in normal-torqued upper central incisor.

Application of the same force in normal-torqued central incisor led to a palatal movement in apical (-0.000123 mm) and labial displacement in incisal edge (0.0000326 mm) in LiO. In normal-torqued central incisor on application of intrusive force induced a palatal displacement in apical area (-0.000456mm) and a labial movement in the incisal edge (0.000614mm) in LaO (Fig. 5).

**Figure 6:**
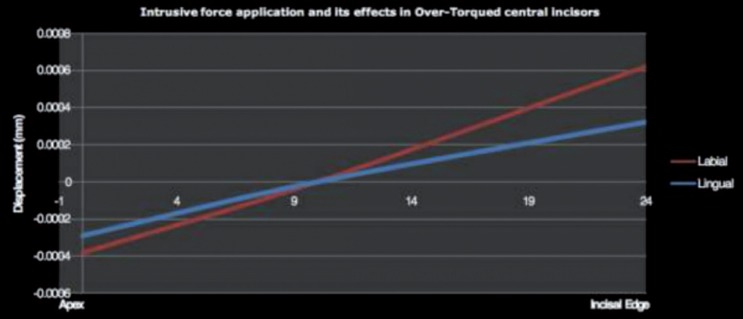
Tooth displacement in over-torqued upper central incisor.

Over-torqued upper central incisor subjected to intrusive force showed labial crown displacement of the incisal edge in LaO (0.000621mm) and LiO (0.000323 mm) (Fig. 6). In Over-torqued upper central incisor the crown displacement labially is more in lao than LiO. Palatal displacement of the apical area is noticed in both techniques, more in LaO (-0.000384mm) than LiO (-0.000292 mm).

## Discussion

The present study was to investigate response of the upper incisor with normal, under and over torque to vertical load in labial and lingual orthodontic system through 3 Dimensional finite element study.

Intrusive forces act differently in both techniques because of the different location of force vectors in relation to the centre of resistance (12). Increasing the vertical intrusive force is one of the methods routinely used to prevent the uncontrolled tipping and obtain bodily type of movement in labial orthodontics (LaO) (13).

Geron *et al.* (14) stated that moments created in LaO are more than LiO, magnitude of crown displacement is more noticeable in LaO. According to Liang *et al.* (15) due to the shorter distance between the force application point and C res, the magnitude of lingual root torque during the intrusive loads is smaller in LiO than LaO.

In this study, in normal, under and high torqued upper central incisors, in both systems a labial crown tipping was observed, labial crown displacement in LaO is more than LiO. The magnitudes of torque loss in intrusive movements in normal, under and high torqued upper central incisors were higher in LaO than LiO.

Results of this study is in accordance with the findings of Jost-Brinkmann *et al.* (16) who claimed that during the vertical loading of upper incisors in LiO, a uniform stress distribution is seen which leads to more predictable tooth movements

According to Jost-Brinkmann *et al.* (16) application of force to a determined transitional point at which pure intrusion moments generated may induce the same results

The findings are also in agreement with previous statement of Shum *et al.* (17) who showed that due to the closer distance of Cres and point of application in LiO during intrusion of a normal or over-torqued upper incisor, lesser moment and labial tipping is expected.

## Conclusions

In LiO, it is critical to control the moment/force ratio, retracting the incisors under mild forces and increasing lingual root torque will play a major key role. Application of the same intrusive forces in labial and lingual systems in upper central incisor with different inclinations showed different responses.

The magnitudes of torque Loss during intrusive loads in incisors with normal, under and over-torque were higher in Labial system than Lingual orthodontic appliances.
